# Expression of minor cartilage collagens and small leucine rich proteoglycans may be relatively reduced in osteoarthritic cartilage

**DOI:** 10.1186/s12891-019-2596-y

**Published:** 2019-05-18

**Authors:** Nobuho Tanaka, Toshiyuki Tashiro, Yozo Katsuragawa, Motoji Sawabe, Hiroshi Furukawa, Naoshi Fukui

**Affiliations:** 1 Clinical Research Center, National Hospital Organization Sagamihara Hospital, 18-1 Sakuradai, Sagamihara, Kanagawa 252-0315 Japan; 2 Department of Orthopaedic Surgery, Tokyo Yamate Medical Center, 3-22-1 Hyakuninncyou, Shinjyuku-ku, Tokyo, 169-0073 Japan; 3 Department of Orthopaedic Surgery, Center Hospital of the National Center for Global Health and Medicine, 1-21-1 Toyama, Shinjyuku-ku, Tokyo, 162-8655 Japan; 4 Department of Molecular Pathology, Graduate School of Health Care Sciences, Tokyo Medical and Dental University, 1-5-45 Yushima, Bunkyo-ku, Tokyo, 113-8510 Japan; 5 Laboratory for Molecular and Genetic Epidemiology, School of Medicine, The University of Tsukuba, 1-1-1 Tennodai, Tsukuba, Ibaraki, 305-8575 Japan; 6 Department of Life Sciences, Graduate School of Arts and Sciences, The University of Tokyo, 3-8-1 Komaba, Meguro-ku, Tokyo, 153-8902 Japan

**Keywords:** Osteoarthritis, Articular cartilage, Collagen, Small leucine rich proteoglycan, Collagen fibril formation, Transmission electron mocroscopy

## Abstract

**Background:**

In osteoarthritis (OA), cartilage matrix is lost despite vigorous chondrocyte anabolism. In this study, we attempted to determine whether altered matrix synthesis is involved in this paradox in disease progression through gene expression analysis and ultrastructural analysis of collagen fibrils within the cartilage matrix.

**Methods:**

Cartilage tissues were obtained from 29 end-stage OA knees and 11 control knees. First, cDNA microarray analysis was performed and the expression of 9 genes involved in collagen fibrillogenesis was compared between OA and control cartilages. Then their expression was investigated in further detail by a quantitative polymerase chain reaction (qPCR) analysis combined with laser capture microdissection. Finally, collagen fibril formation was compared between OA and control cartilage by transmission electron microscopy.

**Results:**

The result of the microarray analysis suggested that the expression of type IX and type XI collagens and fibrillogenesis-related small leucine-rich proteoglycans (SLRPs) may be reduced in OA cartilage relative to the type II collagen expression. The qPCR analysis confirmed these results and further indicated that the relative reduction in the minor collagen and SLRP expression may be more obvious in degenerated areas of OA cartilage. An ultrastructural analysis suggested that thicker collagen fibrils may be formed by OA chondrocytes possibly through reduction in the minor collagen and SLRP expression.

**Conclusions:**

This may be the first study to report the possibility of altered collagen fibrillogenesis in OA cartilage. Disturbance in collagen fibril formation may be a previously unidentified mechanism underlying the loss of cartilage matrix in OA.

**Electronic supplementary material:**

The online version of this article (10.1186/s12891-019-2596-y) contains supplementary material, which is available to authorized users.

## Background

Osteoarthritis (OA) is the most prevalent joint disease in developed countries and primarily affects articular cartilage. In OA, the cartilage matrix is lost gradually, eventually devastating the functional joints. Interestingly, previous studies have repeatedly shown that the chondrocyte metabolism is highly up-regulated within OA cartilage [1–6]. Thus, in OA, the cartilage matrix is lost gradually despite enhanced matrix synthesis. This paradox is often explained by overwhelming catabolism by induced proteinases, but it is also possible that OA chondrocytes may synthesize cartilage matrix of poor quality, which does little to inhibit the loss of the matrix [7]. However, to our knowledge, the latter possibility has never been examined in human OA.

Collagen is the primary component of cartilage matrix and comprises about two-thirds of the dry weight of adult articular cartilage [8]. Collagen forms networks within articular cartilage, which is composed primarily of type II collagen with smaller amounts of type IX and XI collagens [3, 8–10]. In mature articular cartilage, type II collagen comprises more than 90% of cartilage collagen, while type IX and XI collagens comprise only roughly 1 and 3%, respectively [8]. Although minor in quantity, type IX and XI collagens play important roles in the preservation of the collagen network within the cartilage matrix. The importance of type IX and XI collagens for the structural integrity of cartilage matrix has been well demonstrated by the observation of gene engineered mice and humans with genetic diseases [11–15].

Small leucine-rich proteins (SLRPs) are another set of proteins that contribute to the integrity of the collagen network. SLRPs are a family of glycoproteins of small molecular weights which have a characteristic leucine-rich region in the core protein, called the leucine-rich repeat or LRR. Through this region, these proteins exert diverse biological functions [16–19]. Modulation of collagen fibril formation is a known function of SLRPs [20–22]. Since articular cartilage contains several members of this protein family including decorin, biglycan and fibromodulin, changes in their expression may significantly alter the quality of the newly synthesized cartilage matrix.

In this study, we attempted to determine whether matrix synthesis within OA cartilage is affected by the disease through gene expression analyses. To this end, we first conducted a cDNA microarray analysis and compared the expression of nine genes coding cartilage collagens and SLRPs between OA and control cartilages in a zone-to-zone manner. Next, the change in their gene expression was investigated in detail by quantitative polymerase chain reaction (qPCR), paying special attention to the regional differences within the cartilage by use of laser capture microdissection (LCM). Since the results of gene expression analyses indicated possible disturbance in collagen fibril formation within OA cartilage, an ultrastructural analysis was performed using transmission electron microscopy, which indeed demonstrated the appearance of thicker collagen fibrils in OA cartilage.

## Methods

### Cartilage samples

This study was conducted in accordance with the Declaration of Helsinki and was approved by the Institutional Review Committee of the National Hospital Organization Sagamihara Hospital. Prior to the acquisition of cartilage samples, informed consent was obtained in writing from each patient or family of the donor. OA cartilage samples were harvested from 29 end-stage OA knee joints of 29 patients (mean age 72.3 years [range 59–84 years]) who underwent prosthetic surgery within 4 h after the operation. All of the knees were medially involved in the disease, and the diagnosis of OA was based on established criteria for knee OA [23]. Ten knees out of 29 were used for the microarray analysis, 16 for the qPCR analysis combined with LCM, and the remaining 3 for ultrastructural observation.

Control cartilage samples were obtained from 11 non-arthritic knee joints from 11 donors (mean age 81.3 years, range 69–88 years) within 24 h after death. The donors had no known history of joint disease, and the normality of the joint was confirmed macroscopically at the time of harvest. Cartilages from 9 knees were used for the microarray analysis and the qPCR analysis, while the other two were used for ultrastructural observation.

### Laser capture microdissection and quantitative gene expression analyses

For LCM, cartilage tissues were obtained from 16 OA knees and 9 control knees, at 3–5 sites per knee. OA cartilage samples were harvested from both the macroscopically intact areas (preserved areas) and the areas showing various degrees of cartilage degeneration (degenerated areas). At each site, approximately 20 mm × 5 mm of cartilage was obtained in full thickness above the tide mark. The separation of cartilage zones by LCM was performed following a previously described method [5, 24]. In brief, cryosections were prepared from the cartilage tissues in a plane vertical to the joint surface, which were separated into three cartilage zones using an LCM device (PixCell IIe; Arcturus, Mountain View, CA, USA) based on the histological features.

Immediately after LCM, RNA was extracted from the respective cartilage zones using an RNeasy Micro kit (Qiagen GmbH, Hiden, Germany) with routine use of DNase I (Qiagen). cDNA was synthesized using Sensiscript reverse transcriptase (Qiagen). The gene expression was evaluated quantitatively by real-time PCR on a LightCycler (Roche Diagnostics, Basel, Switzerland), using gene-specific primers and probes (Additional file 1: Table S1). SYBR® Premix Ex Taq® Perfect Real Time (Takara Bio, Shiga, Japan) or Premix Ex Taq® Perfect Real Time (Takara Bio) was used for PCR. The cDNA levels were normalized by the expression of *GAPDH*.

### Ultrastructural analyses of cartilage collagen fibrils

For the evaluation of the collagen ultrastructure, cartilage tissue was obtained from the weightbearing areas of the lateral femoral condyles. In OA knees, the tissues were acquired from the preserved areas, after confirming that the areas presented no apparent signs of degeneration in order to minimize the influence of degenerative changes. Transmission electron microscopy (TEM) was performed according to a previously described method [25]. In brief, cartilage tissues were initially immersed in cacodylate butter containing glutaraldehyde which was replaced with cacodylate buffer containing osmium tetroxide. The tissues were then dehydrated and embedded in epon. Sections were cut at 90-nm thickness in a plane vertical to the articular surface, and stained sequentially with 0.1% oolong tea extract (Nissin EM, Tokyo, Japan), aqueous uranyl acetate, and lead citrate. The sections were observed under a JOEL JEM-2000 FX-II electron microscope (Nihon Denshi, Tokyo, Japan). Photomicrographs were taken at a magnification of × 6000.

The quantitative image analysis of collagen fibrils was performed according to a previously described method [25] with some modifications. In detail, two or three photomicrographs were taken on each section, and on each photo, the diameters and density of the collagen fibrils were determined in four 6 μm-square areas along with the percentage of the area occupied by fibrils using an image analysis software program (Image J, version 1.52; NIH, Bethesda, MD, USA). These square were placed in the area within 10 μm from the periphery of the chondrocyte so that newly formed fibrils would be evaluated, avoiding overlapping. Thus, the evaluation was performed over an area of four 6 μm-squares or 144 μm^2^ on each photomicrograph. Two sections were prepared from each cartilage sample, and a total of 15 (OA) and 11 (control) photomicrographs were used for this evaluation.

### Statistical analyses

Statistical differences were determined by the two-tailed *t-*test. R (version 3.3.1 for Windows) was used for calculation, and *p* values < 0.05 were considered to indicate statistical significance.

## Results

### qPCR analyses of the cartilage procollagen gene expression in OA and control cartilage samples

Since the result of cDNA microarray analysis indicated disturbed procollagen expression in OA cartilage (Additional file 2: Figure S1), we investigated their expression in further detail by qPCR analysis combined with LCM. LCM was employed in order to elucidate the regional difference in gene expression precisely, as chondrocyte metabolism is known to differ significantly by the location within cartilage [5, 26–28]. A total of 62 cartilage samples from 16 OA knees and 34 cartilage samples from 9 control knees were used for this analysis.

**Fig. 1:**
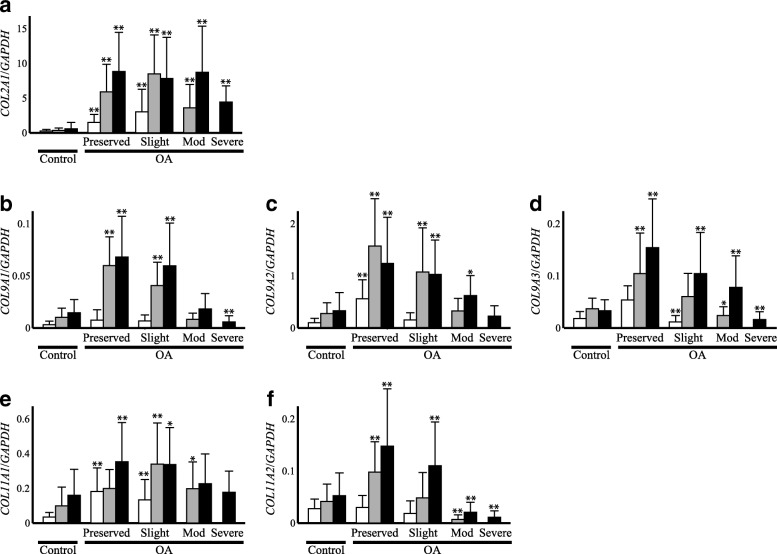
Expression of cartilage procollagen genes in OA (OA) and control cartilage (Control). Cartilage samples were obtained from control or OA knees and precisely divided into superficial, middle and deep cartilage zones using LCM. In each zone, expression of respective procollagen genes was determined by qPCR using *GAPDH* as a housekeeping gene. Since OA cartilage samples were obtained from preserved areas and degenerated areas with various degrees of cartilage degeneration, results are shown for the preserved areas (Preserved), slightly degenerated areas (Slight), moderately degenerated areas (Mod) and severely degenerated areas (Severe). The results of type II (**a**), type IX (**b**-**d**) and type XI procollagen genes (**e** and **f**) are shown. In each graph, open, shaded and solid bars indicate the findings for the superficial, middle and deep cartilage zones, respectively. Bars are the mean + SD of 11–34 samples. *, *p* < 0.05 and **, *p* < 0.01 versus the corresponding zone in control cartilage

As shown by the microarray analysis (Additional file 2: Figure S1A), the expression of *COL2A1* was indeed highly elevated in OA cartilage compared with the control cartilage (Fig. 1a). Although the enhancement was observed in all three cartilage zones, it was most obvious in the preserved areas and slightly degenerated areas, where the expression was increased more than 20-fold. Although still obviously enhanced, the expression of *COL2A1* was somewhat decreased in the middle cartilage zone in moderately degenerated areas and in the deep zone in severely degenerated areas. These were the cartilage zones directly exposed to the joint cavity.

Enhanced expression in OA cartilage indicated by the microarray analysis (Additional file 2: Figure S1, B-D) was also confirmed for all three type IX procollagen genes. The increase was most obvious in the preserved areas and slightly degenerated areas, but the levels of increase for these genes were modest when compared with that of *COL2A1*; the increase in expression for type IX procollagen genes did not exceed 6-fold (Fig. 1b-d). Again, it was noted that the expression of type IX procollagen genes was no longer enhanced, or even reduced in moderately and severely degenerated areas within OA cartilage.

For the type XI procollagen genes, the results of the qPCR analysis were somewhat different from those of the cDNA microarray analysis. Although the microarray data indicated marked increase in their expression in OA cartilage (Additional file 2: Figure S1, E and F), the qPCR analysis revealed that the increase was modest, and limited to the preserved and slightly degenerated areas of OA cartilage. The pattern of expression within OA cartilage differed considerably between the two type XI procollagen genes. For *COL11A1*, the expression did not change markedly throughout all OA cartilage lesions (Fig. 1e). In contrast, the expression of *COL11A2* changed obviously within the OA cartilage; while the expression was enhanced in the middle and deep cartilage zones in the preserved areas, the expression was clearly suppressed in the degenerated areas, according to the degree of cartilage degeneration (Fig. 1f).

### qPCR analyses of the fibrillogenesis-related SLRP gene expression in OA and control cartilage

As suggested by the results of the cDNA microarray analysis (Additional file 3: Figure S2), the expression of SLRP genes in OA cartilage may be increased or reduced compared with that in the control cartilage, depending on the gene, and no common trend was found in the change among the genes. Again, the change in their expression seemed to be rather modest compared with that of the procollagen genes.

**Fig. 2:**
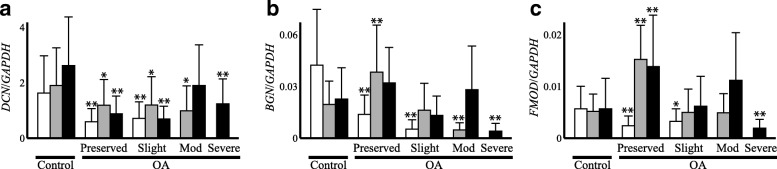
Expression of fibrillogenesis-related SLRP genes in OA (OA) and control cartilage (Control). The expression of decorin (**a**), biglycan (**b**) and fibromodulin (**c**) was determined by qPCR in each of the three cartilage zones of control and OA cartilage, and the results are shown as ratios against *GAPDH* expression as described for Fig. 1. For OA cartilage, Preserved indicates preserved areas, while Slight, Mod and Severe denote slightly, moderately and severely degenerated areas, respectively. In each graph, open, shaded and solid bars indicate results for the superficial, middle and deep cartilage zones, respectively. Bars are the mean + SD of 11–34 samples. *, p < 0.05 and **, p < 0.01 versus the corresponding zone in control cartilage

Consistent with the results of the microarray analysis, the expression of decorin was reduced in OA cartilage compared with that in the control cartilage in all three cartilage zones and across preserved and degenerated areas (Fig. 2a). Within OA cartilage, the expression of decorin did not change markedly among the cartilage zones or between the preserved and degenerated areas, which is another feature of its expression.

Regarding biglycan expression, the results of qPCR analysis markedly differed from those of the microarray analysis. While the microarray indicated a significant increase in the expression in OA cartilage, the qPCR analysis revealed that the expression of biglycan in OA cartilage was similar to or less than the control level (Fig. 2b). Again, among the three SLRP genes, biglycan may be unique in that the expression was reduced in degenerated areas in the cartilage zones exposed to the joint cavity, which is a trend observed for type IX or XI procollagen expression.

The qPCR analysis indicated that the expression of fibromodulin was significantly increased in the preserved areas of OA cartilage in the middle and deep cartilage zones (Fig. 2c). However, such increases in expression were not observed in the degenerated areas, where the expression was similar to or less than that in the control cartilage.

### The comparison of the procollagen gene expression ratio between OA and control cartilage

**Fig. 3:**
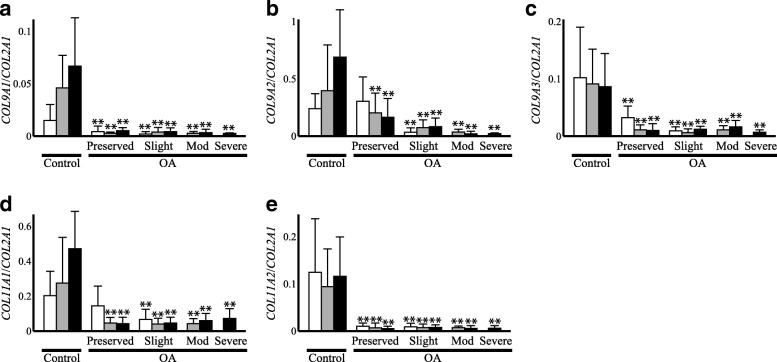
Expression ratio of cartilage procollagen genes in OA (OA) and control cartilage (Control). Based on the results of qPCR analysis, expression ratio of type IX (**a**-**c**) or XI procollagen genes (**d** and **e**) relative to that of type II procollagen gene was compared between OA and control cartilage in a zone-to-zone manner. Results are derived from the data shown in Fig. 1. For OA cartilage, Preserved indicates preserved areas, while Slight, Mod and Severe denote slightly, moderately and severely degenerated areas, respectively. In each graph, open, shaded and solid bars indicate the findings for the superficial, middle and deep cartilage zones, respectively. Bars are the mean + SD of 11–34 samples. **, p < 0.01 versus the corresponding zone in control cartilage

Since the magnitude of change in the expression in OA cartilage differed considerably among the cartilage procollagen genes, we next investigated how the balance in expression changed among the procollagen genes in OA cartilage. First, the expression ratio of type IX to type II procollagen was compared between OA and control cartilage. As anticipated from the results of the qPCR analysis, the expression ratio of type IX to II procollagen was markedly reduced in most parts of the OA cartilage compared with that in the control cartilage (Fig. 3a-c). In the preserved areas of the OA cartilage, the reduction was most apparent with *COL9A1*, followed by *COL9A3*, and least apparent with *COL9A2*. In the degenerated areas, the expression ratio of *COL9A2* to *COL2A1* was reduced compared with that in the preserved areas, while no such trend was noted with *COL9A1* to *COL2A1* or *COL9A3* to *COL2A1*.

Similar to type IX procollagen, the expression ratio of type XI to II procollagen was significantly lower in most parts of the OA cartilage than in the control cartilage (Fig. 3d and e). The reduction was more evident with *COL11A2* than *COL11A1*, with the expression ratio of *COL11A2* to *COL2A1* in OA cartilage being less than 1/10 of that in the control cartilage. For either type XI procollagen gene, the level of reduction was similar throughout the three cartilage zones and across preserved and degenerated areas.

### The comparison of SLRP gene expression ratio relative to type II procollagen between OA and control cartilage

**Fig. 4:**
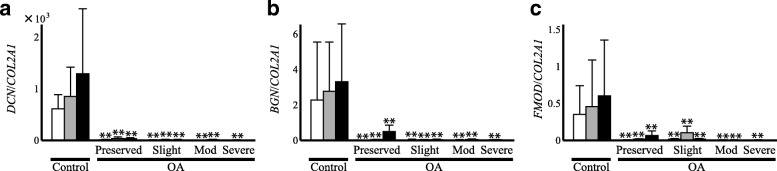
Expression ratio of fibrillogenesis-related SLRP genes relative to that of type II procollagen expression in OA (OA) and control cartilage (Control). Based on the results of qPCR analysis, expression ratio of decorin (**a**), biglycan (**b**) and fibromodulin (**c**) relative to that of type II procollagen was compared between OA and control cartilage in a zone-to-zone manner. Results are derived from the data shown in Figs. 1 and 2. For OA cartilage, Preserved indicates preserved areas, while Slight, Mod and Severe denote slightly, moderately and severely degenerated areas, respectively. In each graph, open, shaded and solid bars indicate the findings for the superficial, middle and deep cartilage zones, respectively. Bars are the mean + SD of 11–34 samples. *, *p* < 0.05 and **, *p* < 0.01 versus the corresponding zone in control cartilage

In the next analysis, the expression ratio of SLRPs relative to type II procollagen was compared between OA and control cartilage. This analysis was performed because the level of SLRP expression can affect the formation of type II collagen fibrils, analogous to type IX or type XI collagen. In the result, the expression ratio of SLRP genes relative to the type II procollagen gene was dramatically reduced in OA cartilage compared with that in control cartilage (Fig. 4a-c). Among the three SLRP genes, the reduction was most obvious with decorin; the expression ratio of decorin in all areas within OA cartilage was less than 1/40 of that in control cartilage (Fig. 4a). Although the ratio was somewhat greater for biglycan and fibromodulin, their expression ratios tended to be reduced in degenerated areas according to the severity of cartilage degeneration (Fig. 4b and c). In moderately and severely degenerated areas, the expression ratio of biglycan or fibromodulin relative to type II procollagen was no more than 1/100 of that in the control cartilage.

### The comparison of collagen ultrastructure between OA and control cartilage

**Fig. 5:**
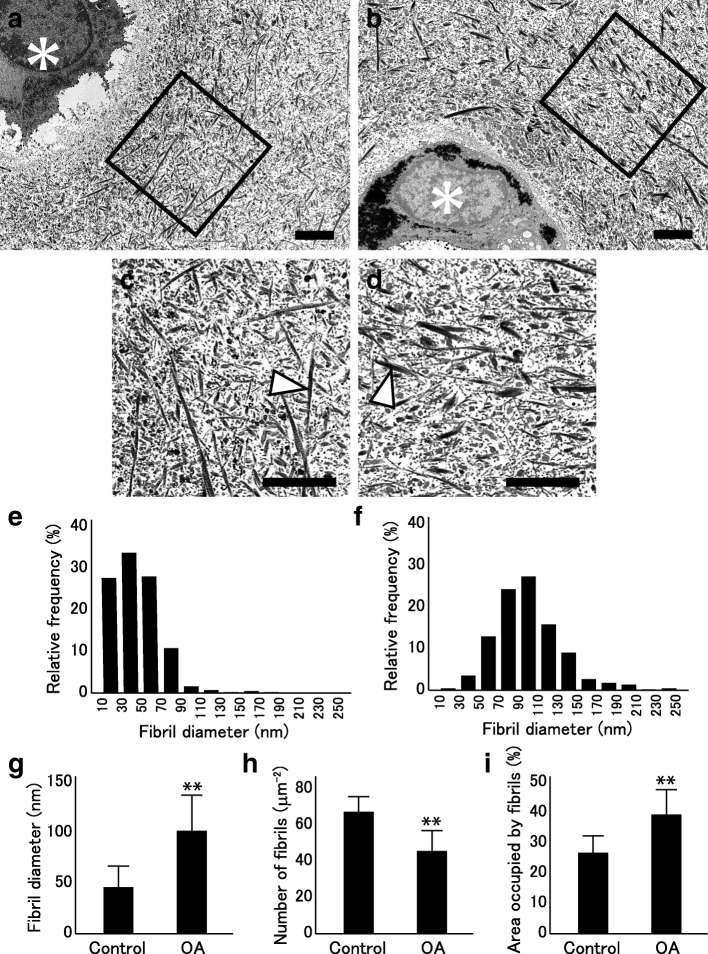
Ultrastructural evaluation of cartilage collagen. Collagen fibrils in control and OA cartilage tissues were observed by transmission electron microscopy. **a** and **b**, Electron photomicrographs of control (**a**) and OA cartilage (**b**). Squares indicate the areas for image analysis, which were placed within 10 μm from the periphery of the chondrocytes. Asterisks indicate chondrocytes. C and D, Higher magnification images of the square areas in control (**c**) and OA cartilage (**d**). Arrowheads indicate representative collagen fibrils. In **a-d**, bars indicate 2 μm. **e** and **f**, Distribution of collagen fibril diameters in control (**e**) and OA cartilage (**f**). **g-i**, Fibril diameter (**g**), number of fibrils per μm^2^ (**h**), and percentage of the area occupied by fibrils (**i**) in control (Control) and OA cartilage (OA). Results are the mean + SD. **, *P* < 0.01 against control

Since the results of gene expression analyses suggested altered collagen fibrillogenesis within OA cartilage, we conducted ultrastructural observation of collagen fibrils in OA and control cartilage by TEM. In control cartilage, collagen fibrils were randomly aligned around chondrocytes with no apparent orientations (Fig. 5a). Image analyses indicated that the diameters of the collagen fibrils largely ranged from 10 to 130 nm (Fig. 5b). In OA cartilage, collagen fibrils were placed randomly in directions around chondrocytes in a manner similar to the control, but the fibrils tended to be thicker and more uneven in diameters (Fig. 5c and d). Thus, the mean fibril diameter in OA cartilage was significantly greater than that in the control cartilage (Fig. 5e). Meanwhile, the density of the collagen fibrils or the number of collagen fibrils per area was reduced in OA cartilage compared with the control cartilage (Fig. 5f). Despite the lower fibril density, the area occupied by collagen fibrils was significantly greater in OA cartilage due to the increase in fibril diameter (Fig. 5g).

## Discussion

In this study, we attempted to speculate the quality of cartilage matrix synthesized by OA chondrocyte through a gene expression analysis. In the analysis, we first found that although the expression of all cartilage procollagen genes was enhanced in OA cartilage, the balance in the expression among the genes was obviously changed compared with control cartilage. Within OA cartilage, the expression of type IX and XI procollagens was reduced relative to that of type II procollagen. For some type IX and XI procollagen genes, such a trend was even more obvious in the degenerated areas of OA cartilage (Fig. 3). Another finding of this study was that the expression of SLRPs that might regulate collagen fibril formation was dramatically altered in OA cartilage relative to the type II collagen expression (Fig. 4).

Previous studies have shown that type IX and XI collagens are minor but essential components of articular cartilage that contribute to the integrity of cartilage matrix. The observation of genetically engineered mice and human hereditary diseases illustrate the importance of type IX and type XI collagen in cartilage integrity [12–14]. In human, a link has been suggested between *COL9A1* and hip OA [11, 15], and several mutations in *COL11A1* or *COL11A2* genes manifest as early-onset OA in the knee and other joints [29–31].

Despite their importance, the expression of type IX and XI collagens in articular cartilage has rarely been investigated. The result of this study has revealed that the expression of these minor collagens is relatively reduced in OA cartilage compared with that of type II collagen. Although we have not investigated the mechanism(s), genetic silencing may be involved in the relative reduction of the minor collagen expression within OA cartilage [32].

In an analogy to type IX and type XI collagens, several members of the SLRP family are known to regulate collagen fibrillogenesis [20–22]. Similar to type IX and type XI procollagen genes, the observations in gene engineered mice demonstrated that cartilage integrity could be impaired by the lack of these SLRPs [33, 34]. In our current analysis, expression of the SLRP genes was enhanced or reduced in OA cartilage depending on the genes and regions within cartilage. Previously, Bock, et al. investigated the expression of decorin and biglycan in human OA cartilage and reported that their expression was most enhanced in the areas adjoining to the main defect [35]. For these genes, our current observation was consistent with their previous observation; the expression of decorin and biglycan showed a trend to be enhanced in the deep zone of moderately degenerated areas (Fig. 2a and b). Since a similar trend is observed for *FMOD* expression (Fig. 2c), enhanced SLRP gene expression in such areas may be a feature of chondrocyte metabolism within OA cartilage.

Taken together, the present findings suggest that collagen fibrillogenesis may be significantly altered in OA cartilage through a deficiency in the minor collagens and SLRPs. Notably, for some genes, such a trend was more obvious in the degenerated areas of OA cartilage, the place where cartilage matrix is being lost by the disease.

In OA cartilage, chondrocyte metabolism undergoes significant changes. Previous studies have shown obvious change in the matrix gene expression [36–38], but the change in the balance among the gene expression has never been investigated. To our knowledge, this study is the first to show that the balance in the expression is dramatically changed in OA cartilage among the matrix components involved in collagen fibrillogenesis. Based on the findings of previous studies, thicker collagen fibrils would be formed in OA cartilage through the reduction in type IX or XI collagen or the SLRPs. This prediction was well supported by the results of our ultrastructural observation: in OA cartilage, the collagen fibrils in pericellular areas were approximately twice as thick as those in the control cartilage (Fig. 5). The appearance of thicker collagen fibrils in human OA cartilage has been reported in several previous studies [39–41], but none of them conducted quantitative evaluations. Our current study may be of particular worth, as we confirmed those previous findings quantitatively, and proposed a possible mechanism underlying the appearance of thicker fibrils in OA cartilage.

Such disturbed fibrillogenesis may be implicated in the loss of cartilage matrix in OA. As mentioned before, the cartilage matrix is lost gradually in OA despite vigorous matrix synthesis. Although this is generally considered to be the result of overwhelming catabolism, our current findings suggest another possible explanation for this paradox in disease progression: in OA cartilage, newly synthesized matrix can be lost readily due to poor matrix integrity. Since degenerated cartilage fragments may induce catabolism within OA joints [42, 43], the synthesis of a fragile cartilage matrix may even result in the promotion of disease progression. Therefore, modification of matrix gene expression could be another strategy for inhibiting the progression of OA.

Obviously, this study has several significant limitations. For example, OA cartilage samples were obtained only from end-stage OA knees, and the mechanisms underlying the altered matrix synthesis were not determined. In addition, the changes in matrix synthesis were estimated only through gene expression analyses and ultrastructural observations, and the change in matrix composition was not analyzed at the protein level. In our qPCR analysis, *GAPDH* was chosen as a housekeeping gene, but the expression of this gene may not be very stable in human articular chondrocytes [44]. Again, the number of control cartilages was limited. Despite these limitations, our current observations convincingly suggest the possibility that altered matrix synthesis may be involved in the loss of the cartilage matrix in OA. Elucidation of the mechanisms underlying this altered matrix synthesis in OA chondrocytes might help to clarify the etiology of OA, a common but still enigmatic disease.

## Conclusions

Although the expression of cartilage procollagens is generally enhanced in OA cartilage, the expression of type IX and type XI procollagens was relatively reduced compared to that of type II procollagen, particularly in degenerated cartilage areas. This trend was more obvious with the expression of decorin, biglycan and fibromodulin, the SLRPs known to be involved in collagen fibril formation. These results suggest that collagen fibrillogenesis may be significantly changed in OA cartilage. This possibility was supported by the result of ultrastructural observation of cartilage collagens, which demonstrated the presence of thicker collagen fibrils within OA cartilage. Thus, the results of current study indicated altered collagen fibrillogenesis within OA cartilage. This could be an unidentified, but potentially critical mechanism underlying the loss of cartilage matrix in OA.

## Additional files

Additional file 1: **Table S1.** Supplementary Table. (DOCX 22 kb)

Additional file 2: **Figure S1.** For the cDNA microarray analysis, cartilage tissues were obtained from 10 OA knees and 9 control knees at the lateral femoral condyles in full thickness above the tidemark, in squares of approximately 10 mm per side. In OA knees, tissues were obtained from areas where no overt sings of cartilage degeneration were observed. Considering that the chondrocyte metabolism differs significantly among cartilage zones, the cartilage tissues were separated into three cartilage zones, and RNA was obtained from each of the zones as previously described (Fukui N, et al. *Arthritis Rheum* 2008;58:3843-53). Using extracted RNA, the gene expression profiles were determined in respective cartilage zones using the Human Gene Expression Microarray (G4112F; Agilent Technologies, Santa Clara, CA, USA), which carries probes for 19,596 human genes. RNA was used for the analysis after confirming that the A260 nm to A280 nm ration was ≤ 1.8, and that the 28S to 18S ration was ≥ 1.4 by an analysis using a Bioanalyzer 2100 with RNA 6000 Nano Chips (Agilent Technologies). All RNA samples were analyzed respectively, yielding 27 sets of microarray data. Using these data, the expression levels of nine cartilage procollagen genes were compared between OA (OA) and control cartilage (Control) in respective cartilage zones, and statistical significance was determined by two-tailed t-test using R (version 3.3.1 for Windows). The level of statistical significance was set at *p* < 0.05. In each graph, open, shaded and solid bars indicate the expression levels in the superficial, middle and deep cartilage zones, respectively. Bars indicate the mean + SD of the signal intensities of 9 (control) or 10 (OA) cartilage samples determined by the microarray analysis. **, *p* < 0.01 versus corresponding zone in control cartilage.. (PDF 348 kb)

Additional file 3: **Figure S2.** Comparison of the expression levels of three SLRP genes in OA (OA) and control cartilage (Control) determined by the cDNA microarray analysis. The expression was compared in respective cartilage zones as described in Additional file 1: Table S1. In each graph, open, shaded and solid bars indicate the findings for the superficial, middle and deep cartilage zones, respectively. Bars indicate the mean + SD of the signal intensities of 9 (control) or 10 (OA) cartilage samples determined by the microarray analysis. *, *p* < 0.05 and **, *p* < 0.01 versus corresponding zone in control cartilage. (PDF 341 kb)

## Abbreviations

LCM: Laser capture microdissection
OA: Osteoarthritis
qPCR: Quantitative polymerase chain reaction
SLRP: Small leucine rich proteoglycan
TEM: Transmission electron microscopy
