# Supplementary material for: Expression of minor cartilage collagens and small leucine rich proteoglycans may be relatively reduced in osteoarthritic cartilage
Source: BMC Musculoskelet Disord. 2019 May 18;20:232. doi: 10.1186/s12891-019-2596-y (PMC6525975; doi:10.1186/s12891-019-2596-y)
Supplement: Supplementary file 1 — Table S1. Supplementary Table. (DOCX 22 kb) [file 12891_2019_2596_MOESM1_ESM.docx]

| **Supplementary Table** | | |  |
| --- | --- | --- | --- |
| **Gene** |  | **Sequence** | **Tm** |
| *COL2A1* | *F:* | 5’-GACATAGGAGGGCCCGAGCA-3’ | 60 |
|  | *R:* | 5’-CGGCACCTGAAGGGAGGTCT-3’ |  |
|  |  |  |  |
| *COL9A1* | *F:* | 5’-CCCTCCTGGAGAGAATGGTT-3’ | 58 |
|  | *R:* | 5’-TCACCTGGGAGACCTATAGC-3’ |  |
|  | *Flu:* | 5’-TGGTGCTCTTGGTTTGAGGGGACC-3’ |  |
|  | *LC:* | 5’-AAAGGTGACTTGGGGAGAAAAGGGGGA-3’ |  |
|  |  |  |  |
| *COL9A2* | *F:* | 5’-GATGCCACTGACCAGCACA-3’ | 58 |
|  | *R:* | 5’-TCCCACGATGCCAGGAAC-3’ |  |
|  |  |  |  |
| *COL9A3* | *F:* | 5’-GCCAGAAGGGCAGCATGG-3’ | 60 |
|  | *R:* | 5’-CCCTCGACTGCCAGACTCTC-3’ |  |
|  | *Flu:* | 5’-TGGGACCCAGTTCTCCTTTATCCCCAG-3’ |  |
|  | *LC:* | 5’-AAGACCGTCGGAACCTGCAATACCCTG-3’ |  |
|  |  |  |  |
| *COL11A1* | *F:* | 5’-TTACTCGGATGGAATGGAAG-3’ | 60 |
|  | *R:* | 5’-GAATCTCCTGAGCAACCTTG-3’ |  |
|  | *Flu:* | 5’-CAGGATGGCTGAGTTGCAGGTCTTTAC-3’ |  |
|  | *LC:* | 5’-AGTTCGGGCTGGATTGGTCTGAGTACC-3’ |  |
|  |  |  |  |
| *COL11A2* | *F:* | 5’-GCCAATGAGGATGAGGTGAG-3’ | 58 |
|  | *R:* | 5’-GTCTGAGAAGGAGGCATCCA-3’ |  |
|  | *Flu:* | 5’-AGACACAGCAAGGCCGGACGGTG-3’ |  |
|  | *LC:* | 5’-TGGAGGTGCGAACGCCTGTGCT-3’ |  |
|  |  |  |  |
| *DCN* | *F:* | 5’-GAGTGGTCCAGTGTTCTGATTTG-3’ | 60 |
|  | *R:* | 5’-ATTCCTTCAGCTGATTCTTGGAC-3’ |  |
|  |  |  |  |
| *BGN* | *F:* | 5’-GTCTGAAGTCTGTGCCCAAAGAGA-3’ | 60 |
|  | *R:* | 5’-ACCAGGTGGTTCTTGGAGATGTAG-3’ |  |
|  |  |  |  |
| *FMOD* | *F:* | 5’- ACAACCAGATCTCACGGGTC-3’ | 58 |
|  | *R:* | 5’- AAGTAGCTATCGGGGACGGT-3’ |  |
|  |  |  |  |
| *GAPDH* | *F:* | 5’-CAGGGACTCCCCAGCAGT-3’ | 60 |
|  | *R:* | 5’-GGGATTGCCCTCAACGACCA-3’ |  |

Primer and probe sequences used for the qPCR analysis. F and R indicate the forward and reverse primer sequences, respectively, while Flu and LC are the sequences of the fluorescein- and LC-Red 640-labeled probes, respectively.
