# Supplementary figures and images for: Expression of minor cartilage collagens and small leucine rich proteoglycans may be relatively reduced in osteoarthritic cartilage
Source: BMC Musculoskelet Disord. 2019 May 18;20:232. doi: 10.1186/s12891-019-2596-y (PMC6525975; doi:10.1186/s12891-019-2596-y)

# Supplementary Figure 1

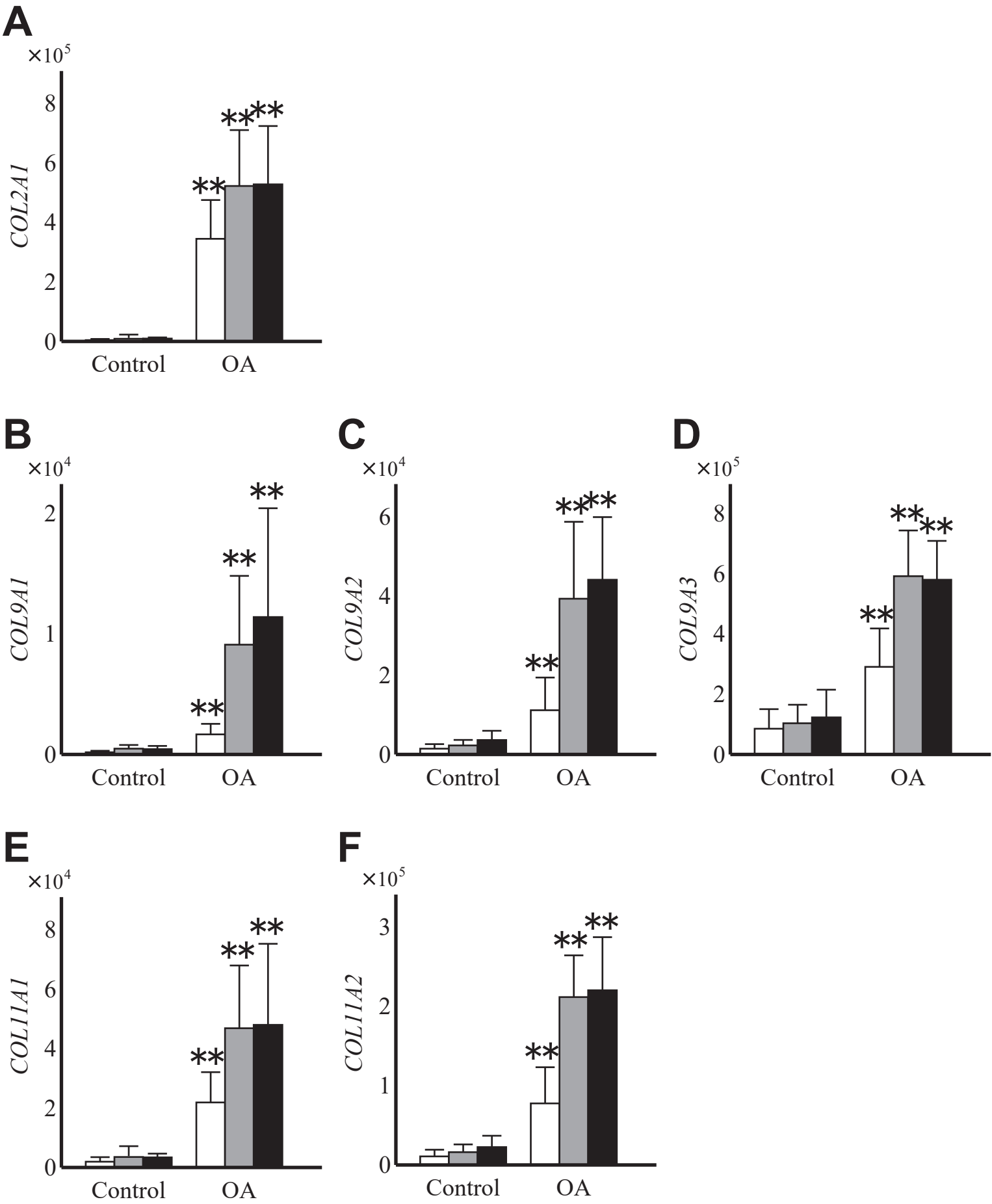

Supplement: Supplementary file 2 — Figure S1. For the cDNA microarray analysis, cartilage tissues were obtained from 10 OA knees and 9 control knees at the lateral femoral condyles in full thickness above the tidemark, in squares of approximately 10 mm per side. In OA knees, tissues were obtained from areas where no overt sings of cartilage degeneration were observed. Considering that the chondrocyte metabolism differs significantly among cartilage zones, the cartilage tissues were separated into three cartilage zones, and RNA was obtained from each of the zones as previously described (Fukui N, et al. Arthritis Rheum 2008;58:3843-53). Using extracted RNA, the gene expression profiles were determined in respective cartilage zones using the Human Gene Expression Microarray (G4112F; Agilent Technologies, Santa Clara, CA, USA), which carries probes for 19,596 human genes. RNA was used for the analysis after confirming that the A260 nm to A280 nm ration was ≤ 1.8, and that the 28S to 18S ration was ≥ 1.4 by an analysis using a Bioanalyzer 2100 with RNA 6000 Nano Chips (Agilent Technologies). All RNA samples were analyzed respectively, yielding 27 sets of microarray data. Using these data, the expression levels of nine cartilage procollagen genes were compared between OA (OA) and control cartilage (Control) in respective cartilage zones, and statistical significance was determined by two-tailed t-test using R (version 3.3.1 for Windows). The level of statistical significance was set at p < 0.05. In each graph, open, shaded and solid bars indicate the expression levels in the superficial, middle and deep cartilage zones, respectively. Bars indicate the mean + SD of the signal intensities of 9 (control) or 10 (OA) cartilage samples determined by the microarray analysis. **, p < 0.01 versus corresponding zone in control cartilage.. (PDF 348 kb) [file 12891_2019_2596_MOESM2_ESM.pdf]

Supplementary Figure 2

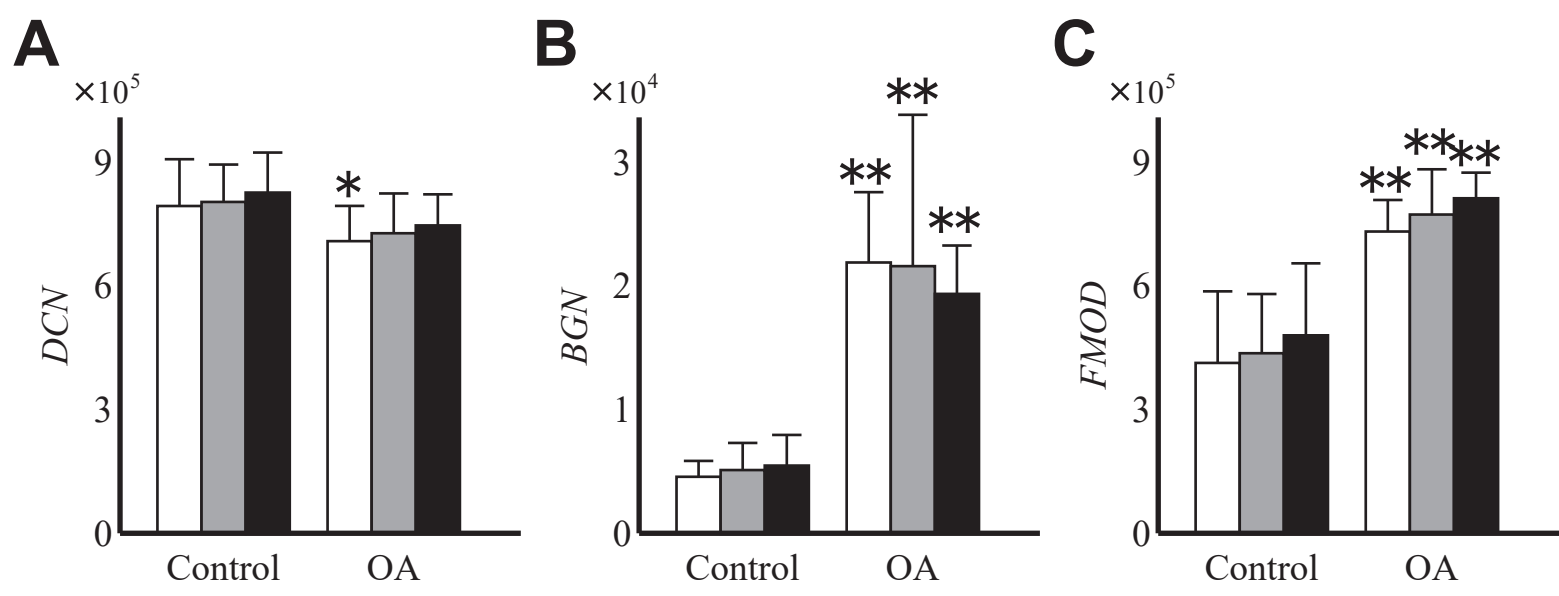

Supplement: Supplementary file 3 — Figure S2. Comparison of the expression levels of three SLRP genes in OA (OA) and control cartilage (Control) determined by the cDNA microarray analysis. The expression was compared in respective cartilage zones as described in Additional file 1: Table S1. In each graph, open, shaded and solid bars indicate the findings for the superficial, middle and deep cartilage zones, respectively. Bars indicate the mean + SD of the signal intensities of 9 (control) or 10 (OA) cartilage samples determined by the microarray analysis. *, p < 0.05 and **, p < 0.01 versus corresponding zone in control cartilage. (PDF 341 kb) [file 12891_2019_2596_MOESM3_ESM.pdf]
